# Cutaneous Neonatal Lupus Erythematosus: A Case Report

**DOI:** 10.7759/cureus.22279

**Published:** 2022-02-16

**Authors:** Sukesh Sukumaran, Deepika Singh

**Affiliations:** 1 Pediatric Rheumatology, Valley Children’s Healthcare, Madera, USA

**Keywords:** rash, antibodies, ana, neonatal lupus, lupus

## Abstract

Neonatal lupus erythematosus (NLE) is a rare autoimmune entity observed in infants born to mothers with autoantibodies against Ro/SSA and La/SSB. Neonatal lupus may present with rash, heart block, hepatic dysfunction, and cytopenia. Although the condition is usually self-limited, serious sequelae can occur.

We present the case of a three-month-old infant who developed multiple erythematous, annular cutaneous lesions at six weeks of age who was ultimately diagnosed with NLE. This case highlights the importance of considering neonatal lupus erythematosus in infants with rashes in the first few months of life. In our case, clinical suspicion and laboratory evaluation helped confirm the diagnosis and facilitate appropriate treatment.

## Introduction

Neonatal lupus erythematosus (NLE) is a rare autoimmune disorder due to the transplacental passage of maternal anti-SSA/Ro and/or anti-SSB/La antibodies [1-3]. Mothers of infants with NLE may have systemic lupus erythematosus, Sjogren syndrome, rheumatoid arthritis, or another autoimmune disorder. However, up to 50% of women who deliver an infant with NLE are completely asymptomatic at the time of delivery [4]. The most common clinical manifestations of NLE include transient cutaneous lesions and permanent heart block. Nearly 10% of infants affected will have both heart block and cutaneous lesions. Other manifestations of NLE, such as hepatobiliary involvement, cytopenia, and central nervous system involvement, have also been reported [1,5-6]. Timely and appropriate evaluation is necessary to prevent complications.

We describe the case of a three-month-old infant born at term who developed a rash at six weeks of life and who was found to have cutaneous neonatal lupus erythematosus. 

## Case presentation

A three-month-old term infant male presented to our institution with multiple erythematous, circular lesions on the face, chest, abdomen, and extremities since the age of six weeks. The rash began as a single lesion on the face, and progressively more lesions appeared. The lesions were not associated with pruritis, fever, cough, or congestion. 

The baby was born via spontaneous vaginal delivery to a 26-year-old primigravida and required stimulation and suctioning after birth. The mother reported that she received regular prenatal care and prenatal labs were negative for human immunodeficiency virus, hepatitis B surface antigen, gonorrhea, chlamydia, and syphilis. She was immune to rubella. The mother was diagnosed with systemic lupus erythematosus 18 months before presentation. She reported compliance with hydroxychloroquine and prednisone but stated she had multiple disease flares throughout her pregnancy. The mother stated that since birth, the baby was breastfeeding well and gaining weight appropriately. There were no sick contacts or animal exposures. 

At the onset of the rash, the baby was evaluated by his primary physician and initially diagnosed with allergic contact dermatitis and later with tinea corporis. However, due to failure to improve despite griseofulvin and topical antifungal agents, he was referred to our institution for further evaluation. 

**Figure 1:**
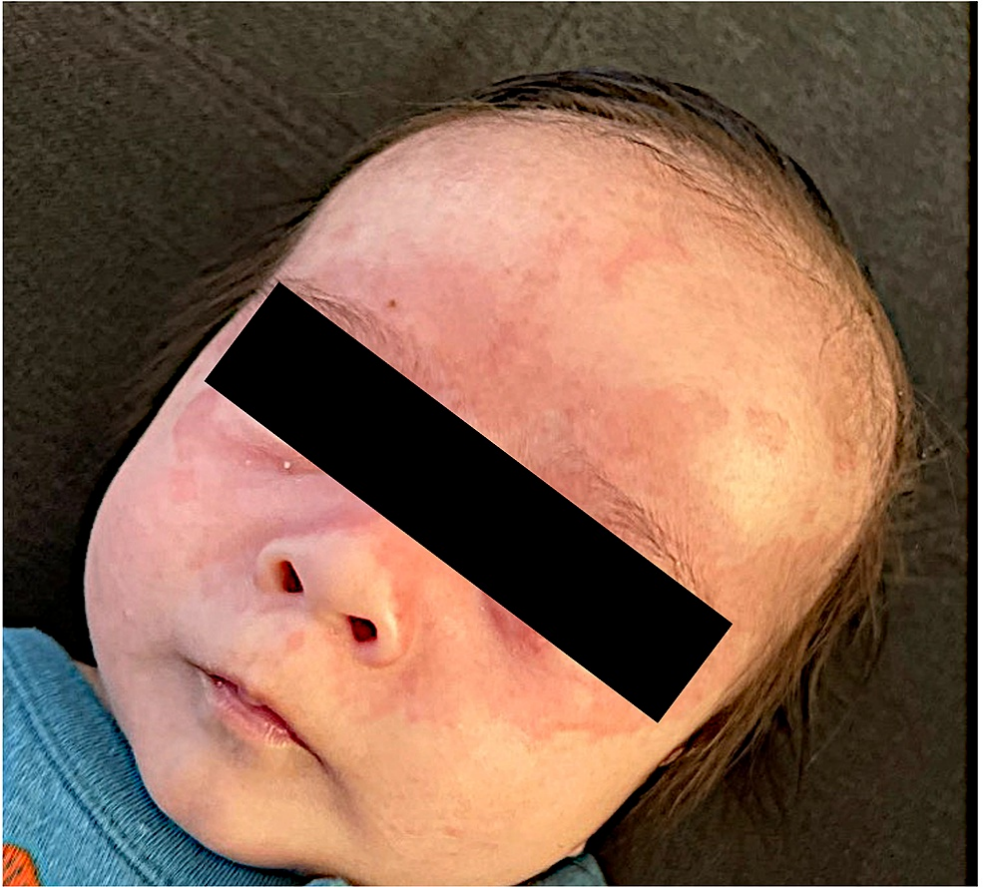
Erythematous, confluent rash on the face, predominantly in the periorbicular area.

**Figure 2:**
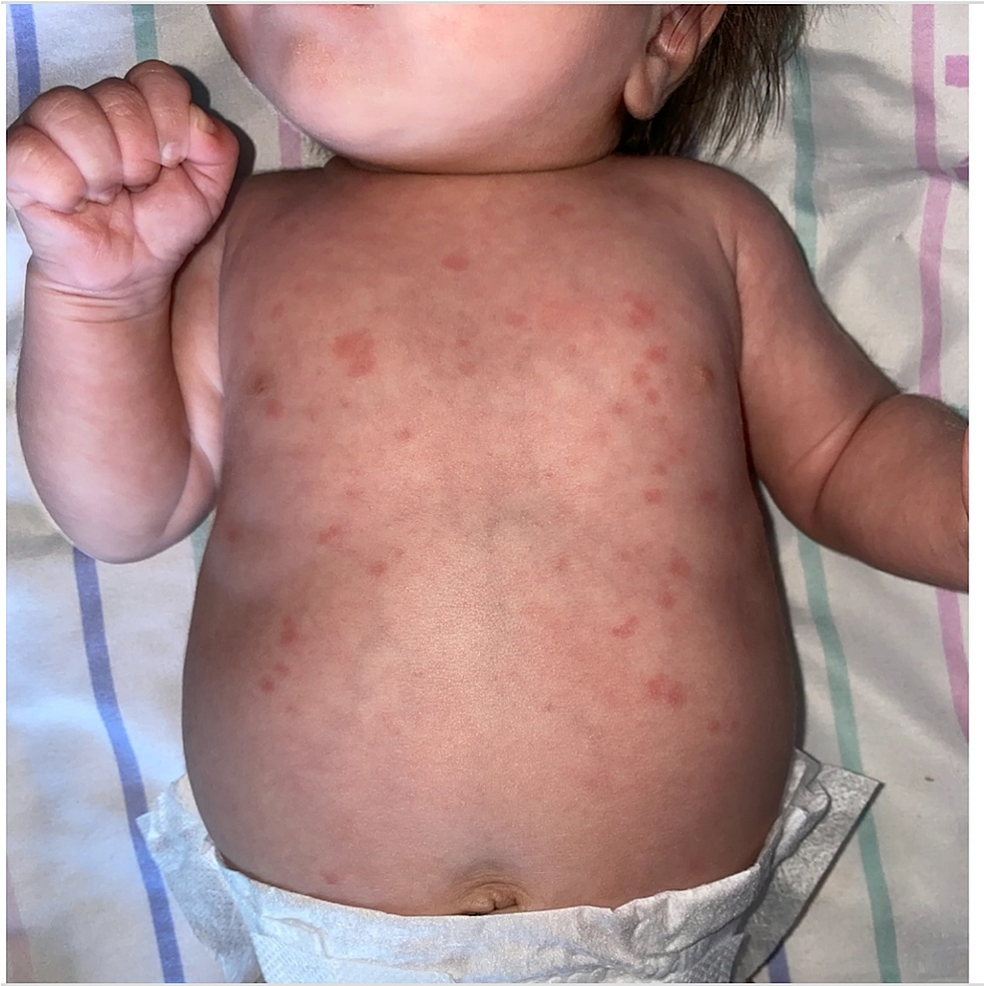
Circular, erythematous lesions on the chest and abdomen of the infant.

Physical exam revealed a term, well-appearing male infant with a temperature of 37°C, heart rate of 106 beats per minute, respiratory rate of 28 breaths per minute, blood pressure of 64/32 mm. On examination of the skin, multiple annular, scaly, erythematous lesions were noted over the face, scalp, chest, and abdomen (Figures 1, 2). Lungs were clear to auscultation, and cardiovascular examination demonstrated normal S1 and S2 with regular rate and rhythm and no murmurs. There was no hepatomegaly. The remainder of his examination was normal.

Laboratory findings on the baby at presentation demonstrated a positive antinuclear antibody (ANA) 1:640 homogenous pattern. The baby had a normal complete blood count and normal aspartate aminotransferase (AST); however, his alanine aminotransferase (ALT) was slightly elevated at 61U/L (range 5-33U/L). His echocardiogram and electrocardiogram were normal. The mother’s antinuclear antibody (ANA) was elevated, and both the newborn and his mother had positive anti-Sjögren’s syndrome-related antigen A (Anti-SSA/Ro) and anti-Sjögren’s syndrome-related antigen B (anti-SSB/La).

Based on the mother’s and the baby’s positive SS-A and SS-B antibodies and the appearance of the rash, the infant was diagnosed with neonatal cutaneous lupus erythematosus. The baby was treated with 0.5% hydrocortisone, and the mother was counseled to avoid sun exposure of the infant. The baby has been slowly improving after the initiation of treatment, and by his six-month follow-up visit, the lesions had completely resolved without scarring, and his liver function tests had normalized. 

## Discussion

NLE is a clinical disease characterized by cardiac, hepatic, dermatological, and hematological involvement in infants with maternal antibodies, including anti-SSA/Ro and/or anti-SSB/La antibodies. The incidence of NLE is thought to be one in 20,000 live births, and females are preferentially affected when compared to males 2:1, but the manifestation of congenital heart block affects both males and females equally [1-4].

Cutaneous lesions are the most common presentation of the disease. The lesions may be found at birth but usually appear within the first few weeks of life. The rash can appear at any site but is most commonly seen on the scalp, face, neck. It is classically periorbital in distribution and can give a "raccoon-eye" like appearance. The rash is frequently erythematous and annular with or without central scaling. However, the rash may also be polycystic plaques, urticarial, ulcerative, or bullous in appearance [1,2,7]. Sun exposure is not necessary for the development of rash, although UV exposure can exacerbate and even induce new lesions. Wisuthsarewong et al. found that infants with NLE may also present with petechiae, persistent cutis marmorata, and discoid lesions [8]. 

The diagnosis of NLE is based on the classic clinical features and the presence of SS-A and SS-B antibodies in the serum of the infant or the mother. As the disease can mimic other conditions, it is essential to keep a broad differential. The differential diagnosis of NLE includes seborrheic dermatitis, urticaria, atopic dermatitis, neonatal acne, tinea corporis, granuloma annulare, erythema multiforme, Langerhans cell histiocytosis, and congenital syphilis [1,2,7]. 

Neonates with NLE should be evaluated by a rheumatologist as a multidisciplinary team may be needed for infants with the extracutaneous disease. Infants with heart block require close monitoring by a cardiologist as most of these infants will require a pacemaker. Infants with NLE should avoid sun exposure and use sunscreen and protective clothing to prevent scarring when exposed to the sun. Topical steroids are beneficial, and most often, immunosuppressants and systemic corticosteroids are not necessary. Infants with cytopenia, hepatic disease, and neurological manifestations may require systemic immunosuppression and intravenous immunoglobulin [1]. 

The prognosis of NLE depends on the organs that are affected. Infants with cutaneous lesions alone have an excellent prognosis, and the rash generally resolves by six months of age with the clearance of maternal antibodies. However, infants with cardiac involvement have high morbidity due to the irreversible nature of the disease. Mortality rates have been reported to be as high as 20%-30% and occur due to congestive heart failure due to congenital heart block. Infants with cytopenia and hepatobiliary disease tend to improve within six months but should be monitored closely for bleeding and liver failure [5,7-8]. The risk of recurrence in future pregnancies is 17%-25%. Infants with NLE are likely at risk for autoimmune diseases, but the exact incidence is unknown.

## Conclusions

Neonatal lupus erythematosus is a clinical condition that results from the transplacental passage of maternal anti-SSA/Ro and/or anti-SSB/La antibodies. Although cutaneous involvement is the most frequent manifestation of NLE, extracutaneous manifestations include congenital heart block, hepatobiliary involvement, neurological involvement, and cytopenia. Most of these tend to disappear spontaneously with the clearance of maternal antibodies from the infant circulation. Cardiac symptoms, however, are not self-resolving and often associated with high morbidity and mortality. Our case underscores the importance of considering NLE when evaluating infants with cutaneous lesions in the postnatal period as delays in diagnosis of NLE are frequent. Although most of the clinical presentations of NLE are self-resolving, prompt evaluation for congenital heart block and evaluation to rule out other systemic involvement is essential to guide further management and prevent adverse outcomes. 

## References

[1] Silverman ED, Laxer RM (1997). Neonatal lupus erythematosus. Rheum Dis Clin North Am.

[2] Vanoni F, Lava SA, Fossali EF (2017). Neonatal systemic lupus erythematosus syndrome: a comprehensive review. Clin Rev Allergy Immunol.

[3] Franco HL, Weston WL, Peebles C. (1981). Autoantibodies directed against sicca syndrome antigens in the neonatal lupus syndrome. J Am Aca Derm.

[4] Cimaz R, Spence DL, Hornberger L, Silverman ED (2003). Incidence and spectrum of neonatal lupus erythematosus: a prospective study of infants born to mothers with anti-Ro autoantibodies. J Pediatr.

[5] Lee LA, Sokol RJ, Buyon JP (2002). Hepatobiliary disease in neonatal lupus: prevalence and clinical characteristics in cases enrolled in a national registry. Pediatrics.

[6] Prendiville JS, Cabral DA, Poskitt KJ, Au S, Sargent MA (2003). Central nervous system involvement in neonatal lupus erythematosus. Pediatr Dermatol.

[7] Wisuthsarewong W, Soongswang J, Chantorn R (2011). Neonatal lupus erythematosus: clinical character, investigation, and outcome. Pediatr Dermatol.

[8] McCune AB, Weston WL, Lee LA (1987). Maternal and fetal outcome in neonatal lupus erythematosus. Ann Intern Med.

